# Duration of Perioperative Chemotherapy in Locally Advanced Gastric Cancer: A “Less Is More” Question When ypN0 Is Achieved

**DOI:** 10.3389/fonc.2021.775166

**Published:** 2021-12-01

**Authors:** Zining Liu, Yinkui Wang, Fei Shan, Xiangji Ying, Yan Zhang, Shuangxi Li, Yongning Jia, Rulin Miao, Kan Xue, Zhemin Li, Ziyu Li, Jiafu Ji

**Affiliations:** Key Laboratory of Carcinogenesis and Translational Research (Ministry of Education/Beijing), Gastrointestinal Cancer Center, Peking University Cancer Hospital & Institute, Beijing, China

**Keywords:** gastric cancer, perioperative chemotherapy, lymph node metastasis, duration, decision tree model

## Abstract

**Backgrounds:**

Perioperative chemotherapy (PEC) and neoadjuvant chemotherapy (NAC) have become a vital part of locally advanced gastric cancer (LAGC) treatment, but the optimal duration of PEC has not been studied. The aim of this study was to demonstrate the possibility of duration reduction in PEC in the adjuvant chemotherapy (AC) phase for ypN0 patients.

**Methods:**

We included LAGC patients who achieved ypN0 after NAC in our institution from 2005 to 2018. The risk/benefit of AC and other covariates were majorly measured by overall survival (OS) and progression-free survival (PFS). We developed a survival-tree-based model to determine the optimal PEC duration for ypN0 patients in different classes.

**Results:**

A total of 267 R0 resection patients were included. There were 55 patients who did not receive AC. The 5-year OS was 74.34% in the non-AC group and 83.64% in the AC group with a significant difference (p = 0.012). Multivariate Cox regression revealed that both AC (AC vs. non-AC: HR, 0.49; 95%CI, 0.27–0.88; p = 0.018) and ypT stages (ypT3-4 vs. ypT0-2: HR, 2.00; 95%CI, 1.11–3.59; p = 0.021) were significant protective/risk factors on patients OS and PFS. A decision tree model for OS indicated an optimal four to six cycles of PEC, which was recommended for ypT0-2N0 patients, while a minimum of five PEC cycles was recommended for ypT3-4N0 patients.

**Conclusion:**

AC treatment is still necessary for ypN0. The duration reduction could be applied for the ypT0-2N0 stage patients but may not be suitable for higher ypT stages and beyond. A multicenter-based study is required.

## Introduction

Since the CLASSIC trial, chemotherapy has become a shot in the arm for locally advanced gastric cancer (LAGC) treatment, independent of surgery types (1). In the past 10 years, improved treating patterns, including neoadjuvant chemotherapy (NAC) and perioperative chemotherapy (PEC), were introduced to complement the conventional treatment strategy: adjuvant chemotherapy (AC) following curative surgery (2, 3). As recently reported from the RESOLVE trial, the timely advanced systemic therapy increases the tolerability of chemotherapy and brings patients better survival outcomes (4).

While there could be some extra benefit for PEC comparing to AC, the regimens and the recommended length in these two modalities are almost the same according to various guidelines (5–7). Among limited selections, 5-fluorouracil (5-Fu) and platinum are the cornerstones of most first-line chemotherapy regimens for gastric cancer (GC). For the commonest dual drugs therapy, as recommended by most trials protocols and guidelines, a total of 6 months of 5-Fu plus platinum-based drugs is applied to all LAGC patients whatever the sequence of the surgery. It is believed that inadequate duration of chemotherapy would lead to an increased risk of recurrence resulting in poorer survival outcomes. On the other hand, as the LAGC is not always responsive to regular cytotoxic drugs, costs may outweigh the benefit considering the accumulation of toxicity, increased adverse events (AEs), and decreased quality of life, which finally negate their survival benefit (8, 9). Moreover, as a promising tumor stage can be achieved from NAC, whether AC is still obligatory and the extent to which the PEC can be “sufficient.” There is still a lack of evidence to say the current PEC treatment span is suitable for all LAGC patients (10).

Although the cut-down for the duration of AC has been conditionally justified in several malignancies (11, 12), relevant studies in GC are scarce, and very few concern the PEC therapy. Some studies pointed out AC failed to provide superior survival improvement in R0 resected gastric and esophagogastric junction adenocarcinoma under PEC setting (13, 14). However, our previous analyses did not completely favor their idea according to which AC is always a risk factor for LAGC patients’ survival in patients with NAC treatment (15, 16). Nevertheless, as several retrospective studies advocated the indiscrimination in survival between post-NAC (yp) and neutral stage (17, 18), the strategies may be adapted to variation in yp stage in patients with initial LAGC diagnosis. For patients with surgery first, lymph node metastasis is the most important indicator for AC, and pN0 patients with lower T stage are not required for chemotherapy (19). Similarly, the ypN status had the greatest prognostic value in our previous report according to which we hypothesized that a shorter duration of PEC might be beneficial for low-risk ypN0 patients (10). This idea was challenging in the realm of PEC without strong evidence, since patients were diagnosed with LAGC at the initiation of the treatment.

Therefore, the current study investigates the role of adjuvant chemotherapy in ypN0 patients after NAC and R0 resection. A further aim is to choose the optimal PEC treatment duration for this specific population.

## Methods

### Patients

The data from a prospective database of all patients who started NAC at the Peking University Cancer Hospital and Institute was searched between December 1, 2005, to June 1, 2018.

The inclusion criteria included the following: (1) proven diagnosis of gastric adenocarcinoma by preoperative and confirmed by postoperative pathology; (2) no signs of distant metastasis at first visit; (3) patients had received NAC before curative gastrectomy; (4) patients had medical records of the postoperative treatment process; and (5) no lymph node metastasis was confirmed by postoperative pathological diagnosis (ypN0).

**Figure 1:**
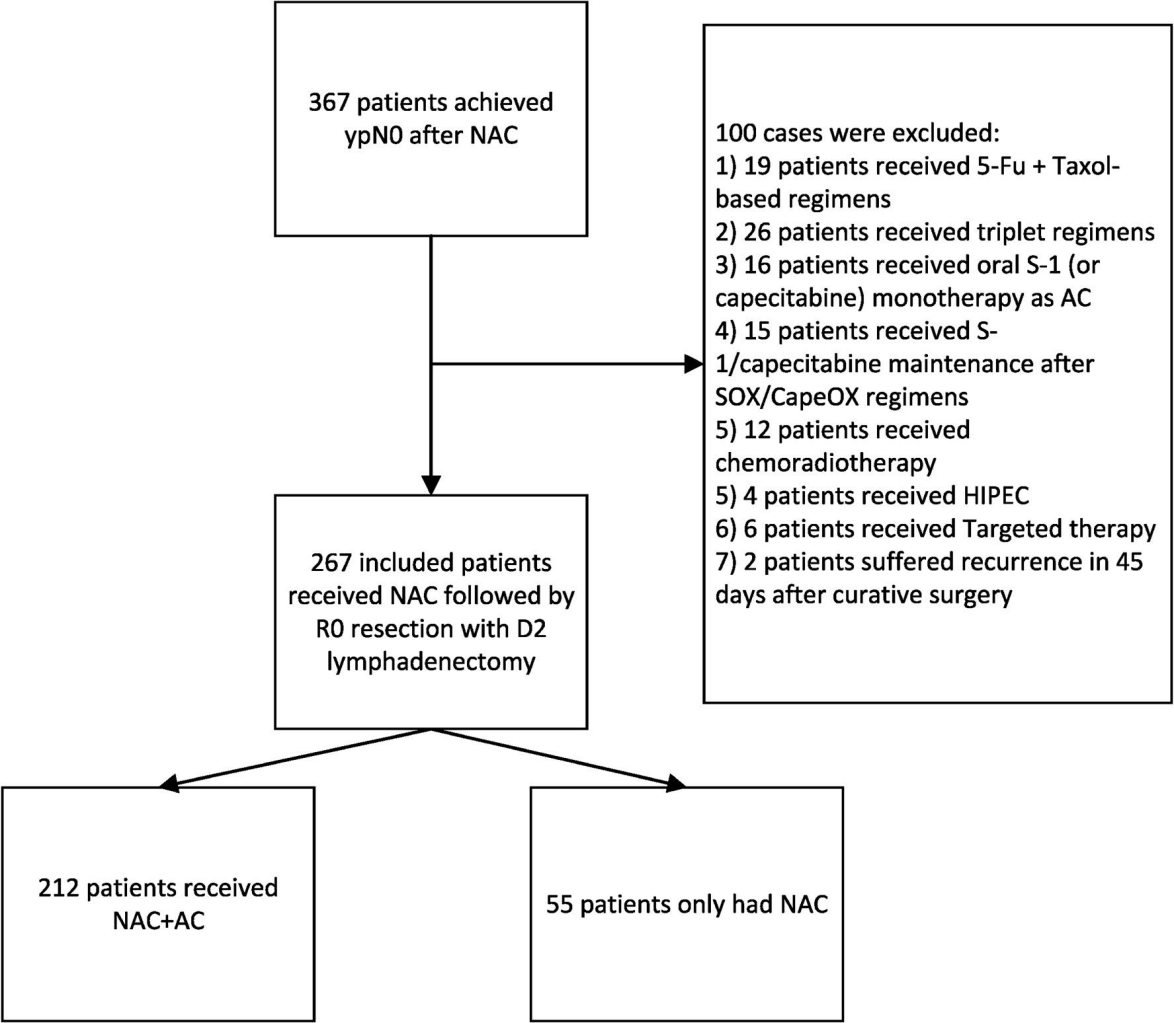
Flowchart showing patient enrolment.

The exclusion criteria were as follows: (1) patients had received chemotherapy regimens other than 5-Fu plus platinum-based doublet regimens or had switched to other regimens during NAC; (2) patients had received radiotherapy or targeted therapy before relapse; (3) patients had received intraperitoneal chemotherapy or hyperthermia intraperitoneal chemotherapy; (4) patients with R1/R2 resection or suffering metastasis 45 days after surgery; (5) patients with D0/D1/D1+ lymphadenectomy; and (6) patients with prior history of gastrointestinal tumor (**Figure 1**). In total, 267 eligible patients were identified in the retrospective database (**Figure 1**).

### Regimen and Radical Surgery

**Figure 2:**
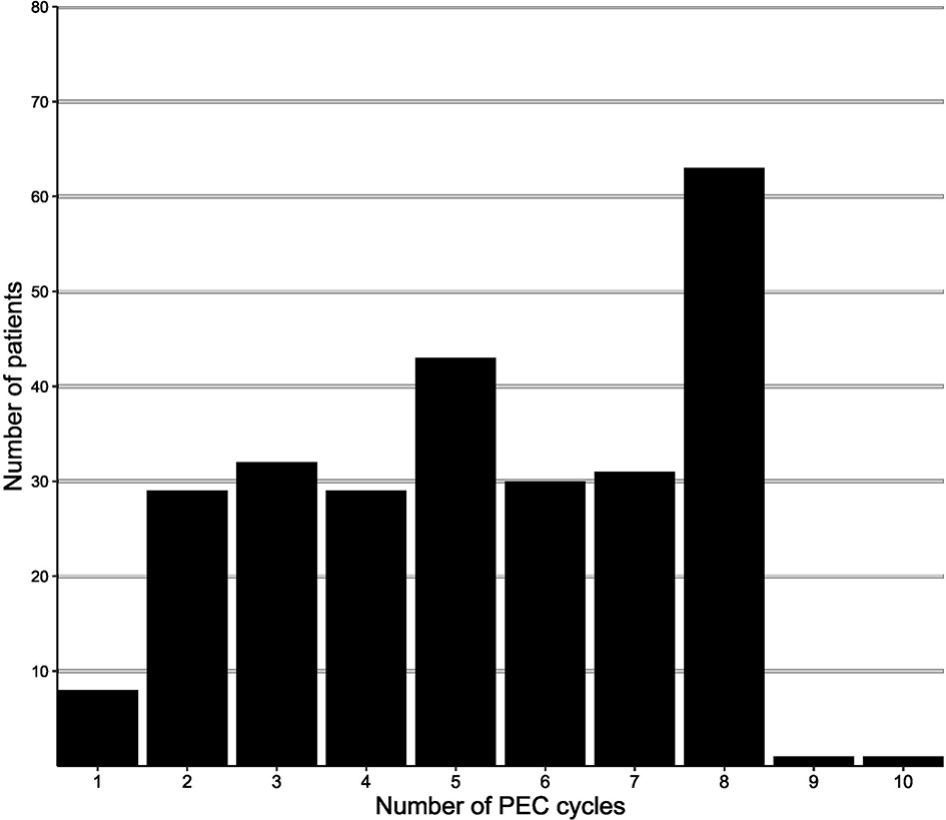
Distribution of PEC duration.

The determination of clinical stages, design for treatment route, preoperative assessment, and prompt intervention for adverse events (AEs) were managed by the multidisciplinary team (MDT). The clinical stages were defined by abdominal computed tomography (CT) scan and/or EUS and/or pre-therapeutic laparoscopic exploration. All patients used platinum plus 5-Fu arms as perioperative regimen, including SOX (oxaliplatin plus S-1), CapeOX (oxaliplatin plus capecitabine), and FOLFOX (oxaliplatin plus 5-Fu/4-Lv). The protocols of each regimen are summarized in **Table 1**. To align the duration of treatment, we regarded three cycles of FOLFOX protocols as two cycles of other 21-day protocols. The distribution of PEC cycles after alignment is shown in **Figure 2**. Dosage reductions occurred if severe adverse events (SAEs) were observed during chemotherapy, as determined by the MDT members. The chemotherapy may be interrupted due to (1) persistent SAE after dosage reduction, (2) patients had poor physical status after surgery resection, (3) the economic conditions did not support following treatment, and (4) patients were unwilling to receive/continue adjuvant chemotherapy after being fully informed. For the preoperative chemotherapy period, the antitumor effect was evaluated using CT scan every two to three cycles. The therapy was prematurely terminated in cases of disease progression, with a curative gastrectomy being immediately performed. Otherwise, gastrectomy or continued NAC was considered after obtaining informed consent and approval from each patient. Subtotal or total gastrectomy plus D2 lymphadenectomy was performed according to the Japanese Gastric Cancer Association (JGCA) guidelines (20).

**Table 1:** Dosage and schedule of the treatment regimen.

Regimen	Drug dosage	Schedule	Duration
SOX	Oxaliplatin: 130 mg/m^2^ IV	Days 1	Q3wk, up to 8 cycles
	S-1: 80 mg (<1.25 m^2^); 100 mg (1.25–1.5 m^2^); 120 mg (>1.5 m^2^) PO	Days 1–14	
CapeOX	Oxaliplatin: 130 mg/m^2^ IV	Days 1	Q3wk, up to 8 cycles
	Capecitabine: 1,000 mg/m^2^ PO	Days 1–14	
FOLFOX	Oxaliplatin: 85 mg/m^2^ IV	Days 1	Q2wk, up to 12 cycles
	Leucovorin: 400 mg/m^2^ IV	Days 1	
	5-Fu: 400 mg/m^2^ IVP	Days 1	
	5-Fu (continuous): 2,400–3,000 mg/m^2^ IV	Days 1–2	

PO, by oral; IV, intravenous.

### Data Collection

The patient characteristics, including age, body mass index (BMI), gender, American Society of Anesthesiologists score (ASA), Eastern Cooperative Oncology Group (ECOG) performance status, tumor location, tumor diameter, histological type, differentiation grade, lymphovascular invasion (LVI), posttherapy pathological (yp) TNM stage according to the 8th American Joint Committee on Cancer (AJCC) guidelines, type of gastrectomy, postoperative complications graded by Clavien–Dindo criteria, adverse event in PEC according to the Common Terminology for Adverse Events (CTCAE) version 4.0., and duration of NAC, AC, and total span of PEC were all recorded (21–23). The overweight threshold was defined as patients with BMI >23.9kg/m^2^ based on the Chinese population (24). All pathological examinations were undertaken by two experienced gastrointestinal pathologists, who were blinded to the group assignment, according to National Comprehensive Cancer Network (NCCN) guidelines (7)

### Follow-up

Patients were followed up regularly *via* physical examination, radiological examination, endoscopic examination, and laboratory examination or telephone call when visits were not possible. These examinations were performed quarterly during the first 2 years, then every 6 months until the fifth year. After 5 years, consultation and follow-ups occurred annually.

### Statistical Analysis

Continuous variables were summarized as mean ± standard deviation or median (IQR) and were compared across groups using the Wilcoxon rank-sum or Kruskal–Wallis test for two or more group comparisons for continuous variables. Categorical variables were analyzed using the chi-squared test or Fisher’s exact test. The relationships between clinical and pathological factors and long-term progression-free survival (PFS) and overall survival (OS) were assessed using univariate log-rank tests. Univariate and multivariate Cox regression analysis was applied to identify the prognostic factors of OS and PFS. Tumor or treatment characteristics that achieved a p < 0.20 in univariate analysis were included in the multivariate analysis. The decision tree classification model was developed using the “rpart” (https://CRAN.R-project.org/package=rpart), with parameters minsplit = 30, cp = 0.000001, and maxdepth = 10, and “rpart.plot” package (https://CRAN.R-project.org/package=rpart.plot). We then selected the complexity parameter (cp) for pruning the tree, which has the lowest 10-fold cross-validation error. We used the restricted cubic spline model to further assess the potential non-linear association between the cycles of PEC and other important covariates based on the result of the decision tree model. The overall and non-linear associations were interpreted by the Wald chi-square test using “rms” package (25). Testing for trends can be applied based on various statistical hypotheses when necessary. For all analyses, p < 0.05 was considered statistically significant. All data were analyzed using R package (version 3.6.2).

## Results

### Patients Characteristics

The selection flowchart is displayed in **Figure 1**. A total of 267 NAC patients achieved ypN0 diagnosis. There were 212 patients who received AC, while 55 patients did not receive AC after curative surgery. Comparing with AC group, patients in the non-AC group had higher age (p < 0.001), poorer physical status (p = 0.004), more comorbidities (p = 0.078), and fewer NAC cycles (p = 0.007). The pathological features of adenocarcinoma were similar between these groups, including tumor size, ypT stage, pathological subtype, and differentiation grade. The demographic and histopathological features have been summarized in **Table 2**.

**Table 2:** Demographic and clinicopathological characteristics in non-AC and AC groups.

N	Overall	Non-AC	AC	p-value
	267	55	212	
Age (years, median [IQR])	61.00 [53.00, 67.00]	68.00 [59.00, 71.00]	59.00 [52.00, 66.00]	<0.001
Sex (%)				0.098
Male	198 (74.16)	36 (65.45)	162 (76.42)	
Female	69 (25.84)	19 (34.55)	50 (23.58)	
BMI (kg/m^2^, median [IQR])	23.51 ± 3.33	23.14 ± 3.55	23.60 ± 3.27	0.366
ECOG (%)				0.004
0	195 (73.03)	30 (54.55)	165 (77.83)	
1	59 (22.10)	20 (36.36)	39 (18.40)	
2	12 (4.49)	5 (9.09)	7 (3.30)	
3	1 (0.37)	0 (0.00)	1 (0.47)	
ASA (%)				0.267
1	33 (12.36)	7 (12.73)	26 (12.26)	
2	206 (77.15)	39 (70.91)	167 (78.77)	
3	28 (10.49)	9 (16.36)	19 (8.96)	
Comorbidity (%)				0.078
No	195 (73.03)	35 (63.64)	160 (75.47)	
Yes	72 (26.97)	20 (36.36)	52 (24.53)	
Short axis (cm, median [IQR])	2.00 [1.00, 3.00]	2.00 [1.00, 3.00]	2.00 [1.00, 2.50]	0.278
Long axis (cm, median [IQR])	2.50 [1.50, 3.50]	3.00 [2.00, 3.50]	2.50 [1.50, 3.52]	0.521
Location (%)				0.072
Upper	88 (32.96)	26 (47.27)	61 (28.91)	
Middle	38 (14.23)	5 (9.09)	33 (15.64)	
Distal	136 (50.94)	23 (41.82)	113 (53.55)	
Diffused	5 (1.87)	1 (1.82)	4 (1.90)	
Location				0.010
Proximal	180 (67.42)	26 (47.27)	61 (28.91)	
Distal	87 (32.58)	29 (52.73)	150 (71.09)	
ypT stage				0.558
ypT0	41 (15.36)	8 (14.55)	33 (15.57)	
ypT1a	24 (8.99)	4 (7.27)	20 (9.43)	
ypT1b	27 (10.11)	7 (12.73)	20 (9.43)	
ypT2	55 (20.60)	10 (18.18)	45 (21.23)	
ypT3	47 (17.60)	6 (10.91)	41 (19.34)	
ypT4a	67 (25.09)	18 (32.73)	49 (23.11)	
ypT4b	6 (2.25)	2 (3.64)	4 (1.89)	
cN status				0.924
cN0	57 (21.35)	12 (21.82)	45 (21.23)	
cN+	210 (78.65)	43 (78.18)	167 (78.77)	
Pathology				0.907
Adenocarcinoma	217 (81.27)	45 (81.82)	172 (81.13)	
Mucin/ring cell	50 (18.73)	10 (18.18)	40 (18.87)	
Differentiation				0.908
Well-moderate	94 (35.21)	19 (34.55)	75 (35.38)	
Poor	173 (64.79)	36 (65.45)	137 (64.62)	
Resection type				0.161
Subtotal	172 (64.42)	31 (56.36)	141 (66.51)	
Total	95 (35.58)	24 (43.64)	71 (33.49)	
NAC cycles				0.007
1	23 (8.61)	8 (14.55)	15 (7.08)	
2	96 (35.96)	24 (43.64)	72 (33.96)	
3	124 (46.44)	14 (25.45)	110 (51.89)	
4	21 (7.87)	8 (14.55)	13 (6.13)	
5	2 (0.75)	1 (1.82)	1 (0.47)	
6	1 (0.37)	0 (0.00)	1 (0.47)	
AC cycles				<0.001
0	38 (14.23)	55 (100.00)	0 (0.00)	
1	27 (10.11)	0 (0.00)	38 (17.92)	
2	38 (14.23)	0 (0.00)	27 (12.74)	
3	37 (13.86)	0 (0.00)	38 (17.92)	
4	49 (18.35)	0 (0.00)	37 (17.45)	
5	22 (8.24)	0 (0.00)	49 (23.11)	
6	1 (0.37)	0 (0.00)	22 (10.38)	
8	38 (14.23)	0 (0.00)	1 (0.47)	
PEC cycles				<0.001
1	8 (3.00)	8 (14.55)	0 (0.00)	
2	29 (10.86)	24 (43.64)	5 (2.36)	
3	32 (11.99)	4 (25.45)	18 (8.49)	
4	29 (10.86)	8 (14.55)	21 (9.91)	
5	43 (16.10)	1 (1.82)	42 (19.81)	
6	30 (11.24)	0 (0.00)	30 (14.15)	
7	31 (11.61)	0 (0.00)	31 (14.62)	
8	63 (23.60)	0 (0.00)	63 (29.72)	
9	1 (0.37)	0 (0.00)	1 (0.47)	
10	1 (0.37)	0 (0.00)	1 (0.47)	
NAC regimen (%)				0.001
SOX	130 (48.69)	34 (61.82)	96 (45.28)	
CapeOX	67 (25.09)	3 (5.45)	64 (30.19)	
FOLFOX	70 (26.22)	18 (32.73)	52 (24.53)	
AC regimens (%)				0.201
SOX	95 (44.19)	0 (0.00)	94 (44.34)	
CapeOX	67 (31.16)	0 (0.00)	67 (31.60)	
FOLFOX	53 (24.65)	0 (0.00)	51 (24.06)	
Severe adverse events				0.189
No	211 (79.03)	47 (85.45)	164 (77.36)	
Yes	56 (20.97)	8 (14.55)	48 (22.64)	

Values in parentheses are percentages unless indicated otherwise.

AC, adjuvant chemotherapy; BMI, body mass index; ASA, American Society of Anesthesiologists; ECOG, Eastern Cooperative Oncology Group; NAC, neoadjuvant chemotherapy; SMD, standardized mean difference.

### Adjuvant Chemotherapy Improved Long-Term Outcomes

**Figure 3:**
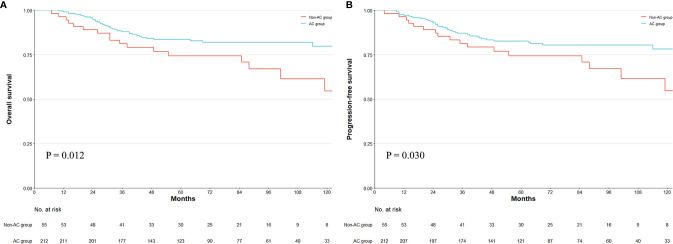
Kaplan–Meier survival plot of overall survival (OS) and progression-free survival (PFS). The survival curve of OS and PFS in whole patients **(A, B)**. Numbers at bottom indicate patients at risk. p-value stands for log-rank test.

Among the entire cohort, 59 patients suffered recurrence, among which 56 patients died of tumor. The median follow-up period among all patients was 75 months (IQR, 29–75 months) estimated by the reverse Kaplan–Meier method. The follow-up time showed no statistical difference between groups (non-AC vs. AC, 87.00 vs. 74.00 months, p = 0.759). Comparing the survival curves for whole patients, the 5-year OS was 74.34% in the non-AC group and 83.64% in the AC group (see **Figure 3A**). The 5-year PFS was 74.45% in the non-AC group and 82.74% in the AC group (**Figure 3B**). The OS and PFS were significantly different between the non-AC and AC groups (log-rank p = 0.012 and p = 0.030, respectively). We used Cox regression to investigate the predictive ability and interaction effects between covariates. In the univariate analyses, female, ECOG (≥2), maximum tumor diameter (≥5cm), total gastrectomy, ypT stage, mucinous or signet ring cell, LVI, and SAE were potential risk factors, while being overweight, AC treatment, and cycles of PEC were protective factors for both OS and PFS (p < 0.20). Considering the PEC cycles confounded with the AC treatment, this factor was exempt from the multivariate analysis. In the multivariate Cox model, the ypT (ypT3-4 vs. ypT0-2: HR, 2.00; 95%CI, 1.11–3.59; p = 0.021), AC treatment (AC vs. non-AC: HR, 0.49; 95%CI, 0.27–0.88; p = 0.018), and LVI (LVI vs. non-LVI: HR, 2.30; 95%CI, 1.00–5.31; p = 0.050) were significant prognostic factors for OS. In the analysis of PFS, the ypT is the only statistically significant prognosticator (ypT3-4 vs. ypT0-2: HR, 2.02; 95%CI, 1.14–3.58; p = 0.016), while AC treatment had a strong tendency towards statistical significance (AC vs. non-AC: HR, 0.56; 95%CI, 0.31–1.00; p = 0.051), followed by mucinous/signet-ring cells (HR, 1.68; 95%CI, 0.94–3.03; p = 0.081), SAE (HR, 1.68; 95%CI, 0.93–3.02; p = 0.086), and LVI (HR, 2.06; 95%CI, 0.90–4.72; p = 0.087) with marginal significance (**Table 3**).

**Table 3:** Univariate and multivariate analyses of prognostic factors.

	OS				PFS			
	Univariate HR	p-value	Multivariate HR	p-value	Univariate HR	p	Multivariate HR	p-value
Age (>60 years)	1.07 (0.63–1.81)	0.805			1.02 (0.61–1.70)	0.954		
Sex (female)	1.67 (0.96–2.90)	0.071	1.30 [0.71–2.36]	0.390	1.53 (0.89–2.65)	0.125	1.17 [0.64–2.11]	0.613
BMI (>23.9 kg/m^2^)	0.621 (0.36–1.08)	0.092	0.64 [0.36–1.14]	0.133	0.62 (0.36–1.06)	0.078	0.63 [0.36–1.10]	0.106
ECOG (>1)	1.87 (0.79–4.39)	0.153	1.91 [0.79–4.63]	0.150	1.75 (0.74–4.10)	0.200	1.72 (0.65–1.91)	0.229
ASA								
1	1.00				1.00			
2	0.83 (0.40–1.73)	0.617			0.89 (0.43–1.85)	0.751		
3	0.84 (0.29–2.41)	0.748			0.98 (0.43–2.24)	0.961		
Comorbidity	1.28 (0.73–2.25)	0.382			1.18 (0.68–2.05)	0.564		
Diameter (cm)	2.09 (1.12–3.90)	0.020	1.02 [0.50–2.08]	0.956	2.23 (1.22–4.07)	0.009	1.16 [0.59–2.30]	0.670
NAC duration (>2 cycles)	1.16 (0.68–2.00)	0.585			1.10 (0.65–1.86)	0.719		
AC administration	0.50 (0.28–0.87)	0.014	0.49 [0.27–0.88]	0.018	0.55 (0.31–0.95)	0.032	0.56 [0.31–1.00]	0.051
Tumor location (distal vs others)	0.95 (0.55–1.66)	0.863			1.02 (0.60–1.75)	0.932		
Total gastrectomy	1.53 (0.90–2.60)	0.115	1.34 [0.77–2.34]	0.305	1.61 (0.96–2.70)	0.068	1.38 [0.81–2.37]	0.234
ypT (per stage increase)	2.32 (1.38–3.88)	0.001			2.28 (1.38–3.76)	0.001		
ypT3–4	2.20 (1.28–3.78)	0.004	2.00 [1.11–3.59]	0.021	2.25 (1.32–3.81)	0.003	2.02 [1.14–3.58]	0.016
cN+ stage	1.05 (0.54–2.04)	0.881			1.12 (0.58–2.17)	0.729		
Poor differentiation	1.12 (0.64–1.95)	0.693			1.12 (0.65–1.92)	0.680		
Mucinous or signet-ring cells	1.83 (1.03–3.28)	0.041	1.57 [0.86–2.88]	0.141	1.91 (1.08–3.36)	0.025	1.68 [0.94–3.03]	0.081
LVI	2.17 (0.98–4.82)	0.057	2.30 [1.00–5.31]	0.050	2.03 (0.92–4.49)	0.081	2.06 [0.90–4.72]	0.087
Severe complications	1.37 (0.65–2.91)	0.410			1.26 (0.60–2.67)	0.539		
SAE	1.55 (0.87–2.78)	0.140	1.63 [0.88–3.01]	0.118	1.60 (0.91–2.83)	0.103	1.68 [0.93–3.02]	0.086
Number of cycles	0.89 (0.79–1.01)	0.069			0.92 (0.82–1.04)	0.203		

Values in parentheses are 95% confidence intervals.

AC, adjuvant chemotherapy; BMI, body mass index; ASA, American Society of Anesthesiologists; ECOG, Eastern Cooperative Oncology Group; HR, hazard ratio; PFS, progression-free survival; OS, overall survival; NAC, neoadjuvant chemotherapy.

### Increased Duration of PEC Had More OS Benefit on ypT3-4 Patients

**Figure 4:**
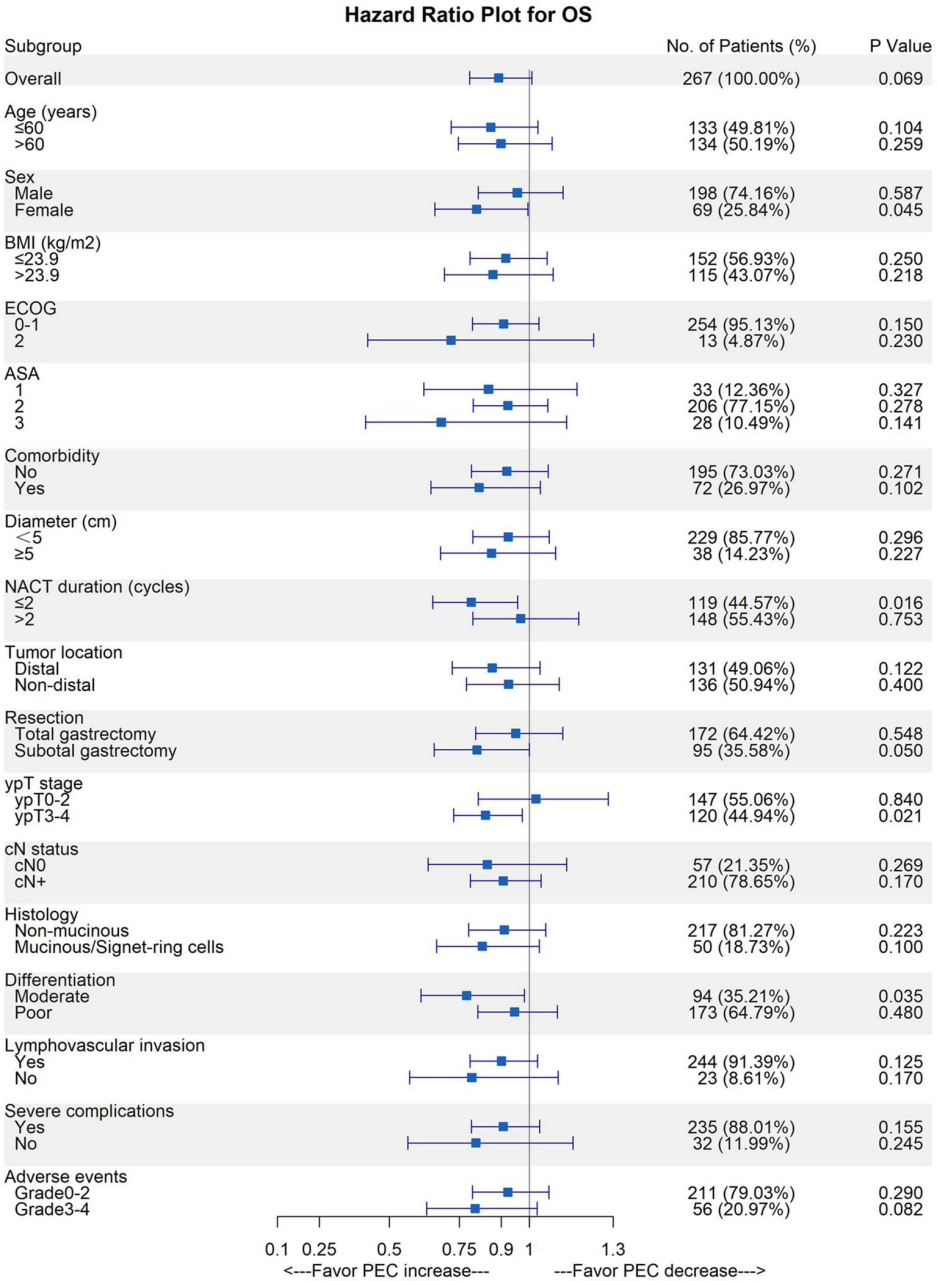
Forest plot for OS benefit from PEC increase in each subgroup.

In the previous context, we discovered the survival benefit of AC administration. With the increase in the PEC cycles, a trend for OS was discovered (per cycle increase: HR, 0.89; 95%CI, 0.79–1.01; p = 0.068). While the simple NAC duration did not bring survival improvement, we assumed the cycles of PEC could influence ypN0 patients’ survival aside from AC existence. We adopted an exploratory subgroup analysis to find the indications for prolonging PEC cycles (**Figure 4**). Except for ypT subgroups, increased PEC cycles achieved a similar protective effect in patients’ OS in any other covariate subgroup. In the ypT subgroups, the opposite effect on OS was observed: increased PEC cycles had significant improvement on patients’ OS in ypT3-4 patients (HR, 0.84; 95%CI, 0.73–0.97; p = 0.021), while no such benefit can be inferred from ypT0-2 patients (HR, 1.02; 95%CI, 0.82–1.28; p = 0.840).

### How Long the Regimen Last, the Decision Tree Model, and Interpretations

**Figure 5:**
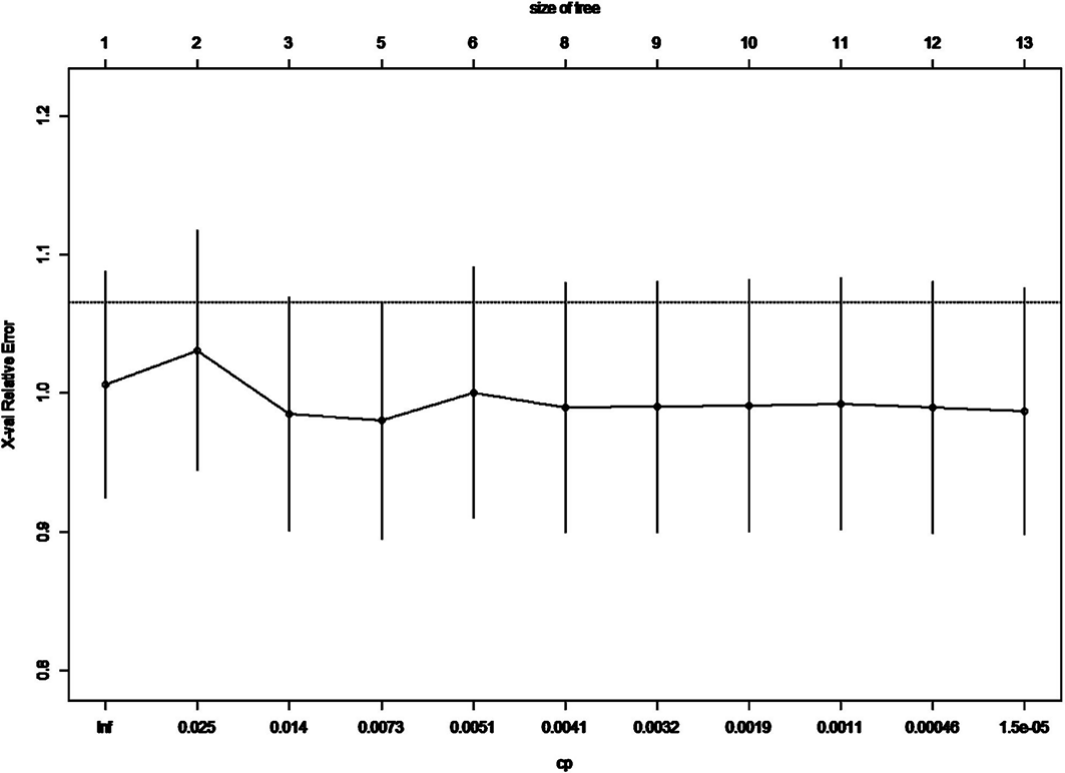
Cross-validation relative error vs. numbers of split and complexity parameter; the lowest CV error rate can be achieved when nsplit reaches 5.

**Figure 6:**
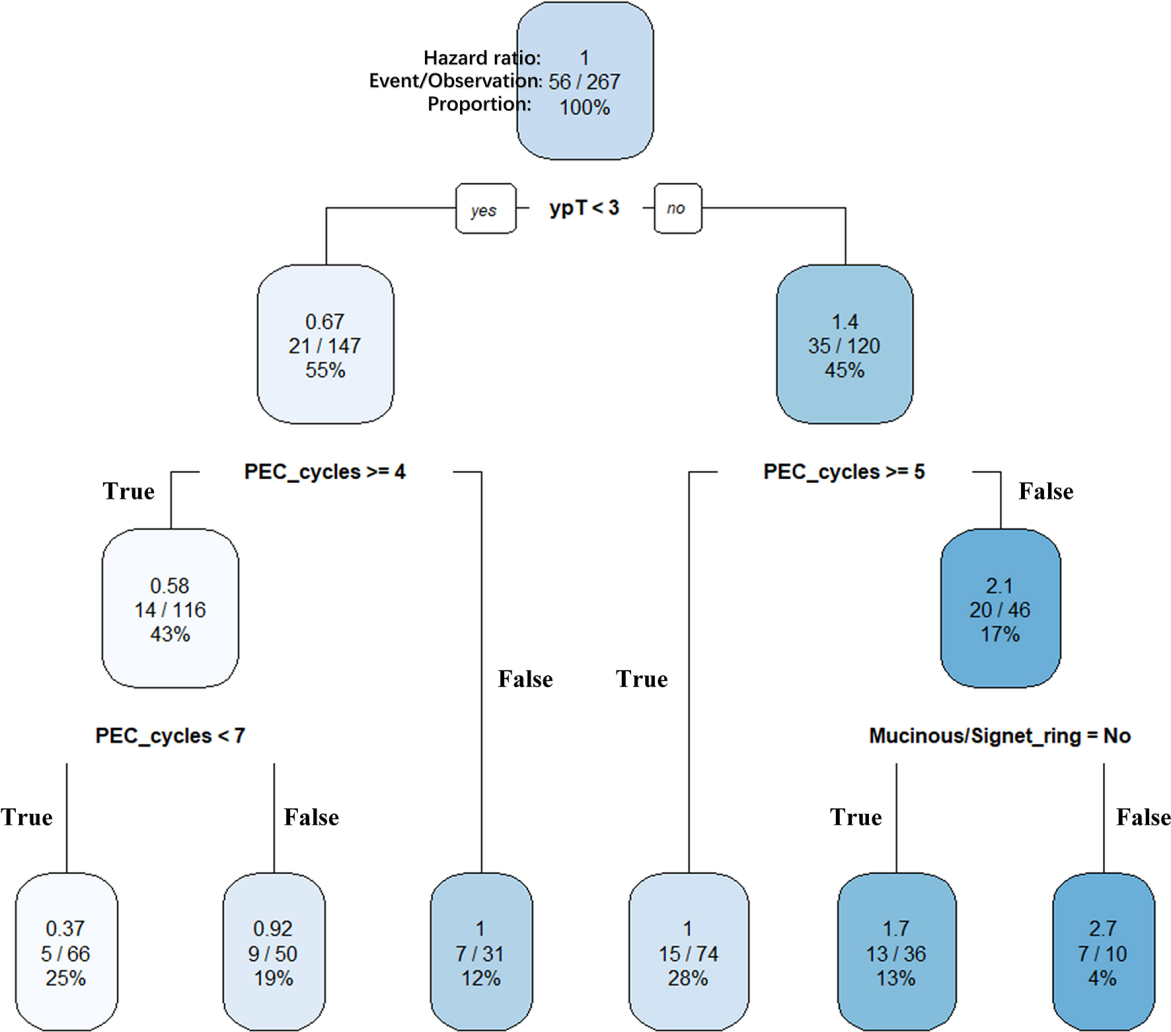
Clinically interpretable decision tree for relative survival risk. ypT (continuous variable from ypT1a to ypT4b), PEC cycles (continuous variable from 1 to 10), and histological type (dichotomous variable) were selected as key features for the final pruned decision tree for overall survival. The predicted hazard ratio in each splitting group using overall samples as reference. Within each internal nodes (conditions), the sub-branch is shown on the left when the condition is True (Yes) and shown on the right when the condition is False (No). The ypT < 3 was set as the root node. Splitting covariate is indicated within each node. The number under each node identifies each subgroup. Six terminal nodes were then identified as follow: node 1, ypT0-2&PEC4-6; node 2, ypT0-2&PEC≥7; node 3, ypT0-2&PEC ≤ 3; node 4, ypT3-4&PEC≥5; node 5, ypT3-4&PEC<5&non-mucinous; and node 6, ypT3-4&PEC<5&mucinous.

**Figure 7:**
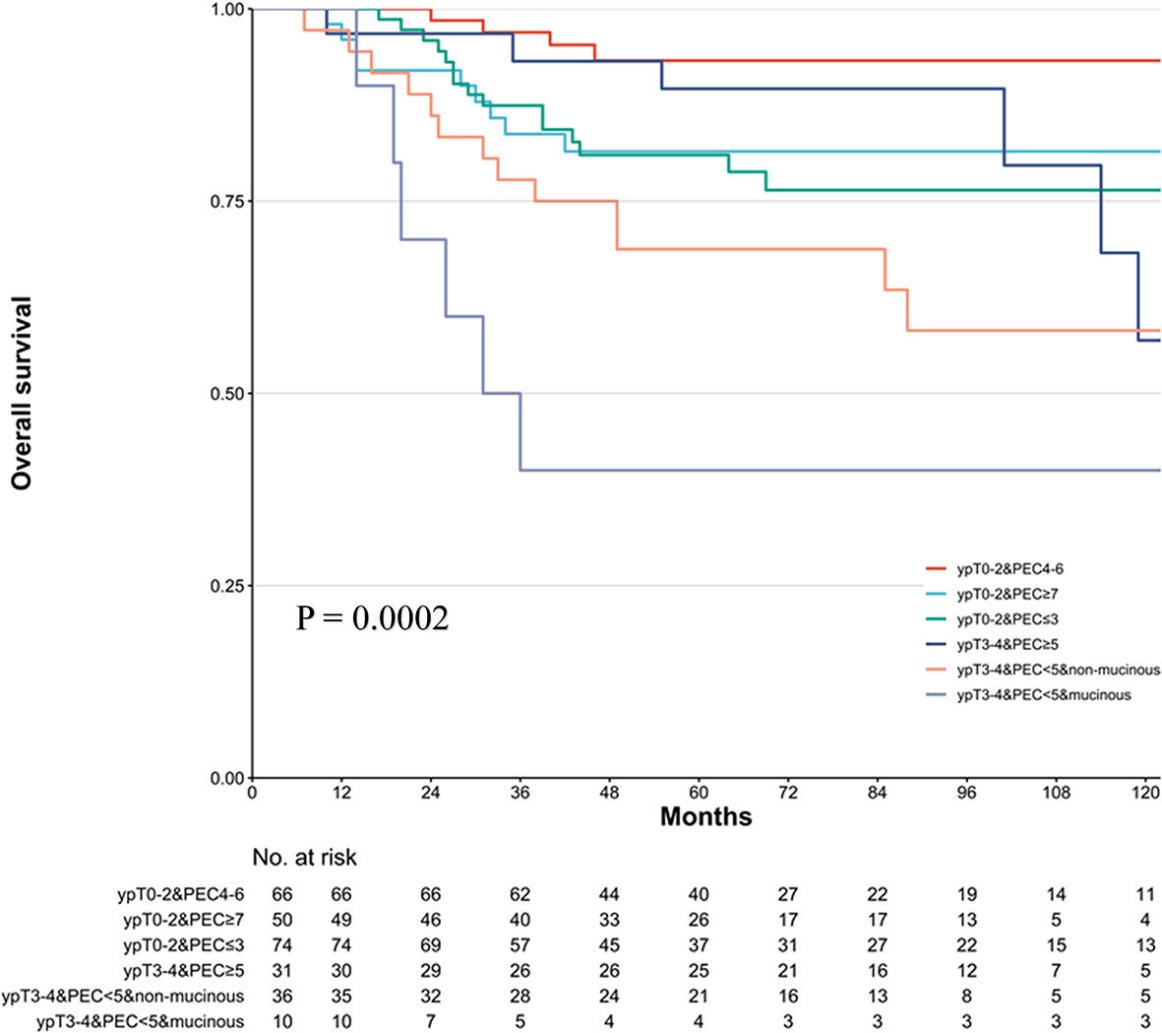
Kaplan–Meier survival plot of the overall survival (OS) in six risk levels classified by decision tree model. p-value stands for log-rank test. The trending log-rank p_trend_ < 0.001.

**Figure 8:**
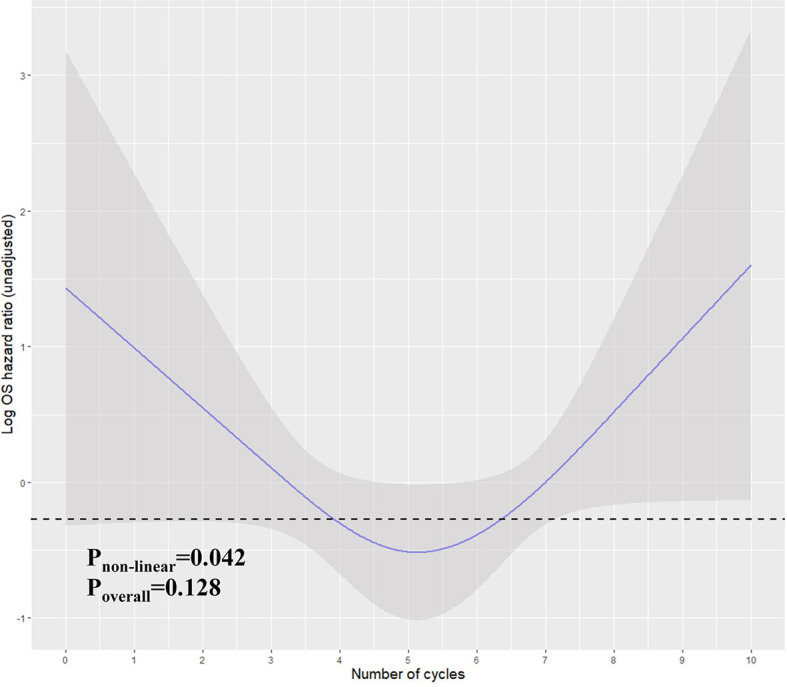
Restricted cubic spline for the unadjusted relationship between duration (cycles) and OS in ypT0-2N0 patients. Y-axis demonstrates the unadjusted log hazard of mortality. The grayed ribbon area reflects bounds of the 95% CI. p-values were for non-linear Wald test. The area under the dashed line indicates the relative HR from PEC cycles 4–6.

In the previous analysis, the ypT classification is the most decisive factor in PEC management. We used the decision tree algorithm to specify the extent of the prognostic value of the PEC cycles and other clinical characteristics on patients’ OS. All significant or marginal significant factors in the multivariate Cox regression were candidates to enter the model. These variables types were listed: ypT stage (continuous variable form T1a to T4b), cycles of PEC (continuous variable), pathological type (dichotomous variable, mucinous/signet-ring vs. normal adenocarcinoma), adverse events grade (dichotomous variable, grade 3–4 vs. grade 0–2), and LVI (dichotomous variable). Tenfold cross-validation for the whole dataset was used to avoid overfitting and give the model better performance. The result indicated that under five times split with six end nodes could the model achieve the least test error (**Figure 5**). The decision tree model was built based on the selection of complexity parameters (**Figure 6**). The KM curves for each end node are shown in **Figure 7** (log-rank p_trend_ < 0.001). The c-index of 3-, 5-, and 10-year OS were 0.688 (95%CI, 0.596–0.782), 0.673 (95%CI, 0.589–0.759), and 0.661 (95%CI, 0.587–0.742), respectively. The decision tree used ypT stage <3 as the root node. For patients with ypT3-4 stage, the cutoff cycles number for PEC was 5, and the mucinous/signet-ring histological type was a sub-decision node that further increase the risk of death, while for patients with ypT0-2 stage, the duration of PEC and the OS benefit were not a simple linear relation: patients with PEC four to six cycles achieve the maximum OS benefit. We used univariate RCS in ypT0-2 patients to investigate the performance of PEC cycles on OS. Similar to the decision tree model, the RCS result indicated that the lowest hazard ratio could be achieved at five to six cycles of PEC with significant non-linear relationship (p = 0.043). However, the overall effect of PEC on OS did not reach a significant level (p = 0.128). The result of RCS partially supported the tree’s algorithm that the benefit of PEC may have a rising-then-falling effect on ypT0-2N0 patients (**Figure 8**).

## Discussion

In this study, we focused on three research topics step-by-step. First, we demonstrated an improved prognosis for AC management. Second, we teased out the relationship between the duration of NAC, AC, and PEC, in which we concluded the more important role that PEC duration played in the treating process. Under the premises, we sought the conditional management of PEC cycles based on our center’s practice, and finally, we demonstrated that a prolonged treatment duration (≥5 cycles) is recommended for ypT3-4N0, while a modest effect with four to six PEC cycles could be more favorable for ypT0-2 patients. Currently, this is the first study in the realm of PEC that the duration of chemotherapy was comprehensively investigated in LAGC.

Conventionally, few pieces of evidence supported the adjuvant chemotherapy administration for GC patients with lower pathological stage, especially for those after receiving D2 lymphadenectomy. Lymph node metastasis is always the key indicator for AC management. For patients who received D2 lymphadenectomy, achieving R0 resection and pN0 diagnosis, pT1-3N0M0 are exempt from the AC according to the 5th JGCA guideline (26), while in the Chinese Society of Clinical Oncology (CSCO) guideline, AC was only recommended for pT3-4N0M0 (5). The newly introduced concept of ypTNM stage has complicated the management for LAGC patients because the “yp” concept has regarded itself as the intermediate product after the NAC treatment (27). Due to the clinical staging methods’ lack of precision, no oncologist can foretell the initially exact tumor stage. When ypN0 achieves, its pretreated N stage can be either N0 or N+, and, logically, the intensity for the postoperative treatment may be varied.

On the other hand, based on our previous report, although there was a potential optimistic estimate for ypT0N0 that had similar survival outcomes to that for ypT1N0 patients, this group of patients is not literally tumor free. Other yp stage classification had similar prognostic indication with pTNM stage (27, 28). Ikoma et al. investigated the survival differences between different ypN statuses and found that the NAC downstaging is not the risk factor for ypN0 patients. No matter the clinical N status before the NAC administration, promising survival outcomes were to be expected (29). Their conclusion has also been confirmed in the current study, and the results drive us to doubt whether there is any additional practical meaning behind the “yp” comparing to “p” TNM stage. Yet, to answer this question is far from easy; the boundary between preoperative and postoperative chemotherapy has already become blurred, since the PEC is now a more common practice in western countries and China compared to surgery with/without AC following. As a result, it is of priority to address the treating strategy in NAC and PEC patients under different conditions. Thus, when ypN0 achieves, a more realistic question should be: to what extent the PEC could reach the maximum benefit and whether AC can be exempt from certain groups of patients.

The decision tree algorithms are effective methods that can deal with mixed continuous and dichotomous covariates. Compared with Cox regression, the tree model results are more easily interpreted and can mimic the clinical decision-making processes. Another advantage of this method is that the cutoff can be exported based on machine learning instead of an arbitrary divide or repeatedly manual attempt. Based on our data, the decision tree model selected ypT < 3 as the root node, which emphasized the ypT strong influence on ypN0 patients’ OS. Under the root decision, the PEC duration had various influences on patients in different ypT groups. In the ypT3-4 branch, the PEC cycles were dichotomized at five, which is easier to understand and can be complemented by the Cox regression, according to which the increase in PEC cycles is accompanied by stable survival gain. In the ypT0-2 branch, however, the tree’s result gave an optimal four to six cycles interval resolution. This indicated that there might be a “dose–response” effect in patients with lower yp stage. The toxicity and the adverse events could overwhelm its antitumor effect in a prolonged chemotherapy administration because the tumor load is already trivial (9). On the other hand, as 88.44% of patients only had one to three cycles of NAC, the conditional benefit of PEC (four to six cycles) justified the AC’s necessity for most ypT0-2N0 patients. We suggest that the early ypTxN0 stage should not be regarded equally as the pTxN0 stage (27). Instead of ypN+, the ypN0 stage is more likely to be concluded as a moderate-to-effective response to NAC, having more remission cases and fewer progression diseases. Moreover, although with relatively low sensitivity, the clinical N+ status measured by either CT or EUS tended to have high specificity and positive predictive value, which means the likelihood of downstaging is high in cases from cN+ to ypN0 (30, 31). Thus, the ypN0 could enrich those chemo-responsive patients to some extent, which warranted the treatment efficacy even for early yp stage patients.

Currently, the duration of the standardized treatment follows the original protocols in phase III trials in which the comparison of treatment length was not routinely designed due to costing and ethical concerns. Sometimes, clinicians lack enough evidence to say that the fixed regimens are optimal, although effective. More importantly, the real-world circumstances are often not as ideal as those in clinical trials (32). Patients may discontinue perioperative or adjuvant treatment due to various reasons, e.g., adverse events, low life quality, and financial burdens (33–35). Because of these, the reduced duration for chemotherapy and other treatments has attracted widespread attention in recent years. In the IDEA collaboration, the 3 months (corresponding to four 21-day cycles) of adjuvant therapy confirmed only 0.4% inferiority versus 6 months (corresponding to eight 21-day cycles) duration in overall survival in patients with stage III colorectal cancer (36). In stage I−II ovarian clear cell carcinomas, Prendergast et al. found that similar survival outcomes could be reached in three- and six-cycle groups (12). This result was prospectively confirmed by the TOSCA phase 3 trial (37). In LAGC, studies focusing on the different duration of adjuvant chemotherapy are few (38, 39), and only our previous research has taken PEC into consideration (10). In the light of these previous studies, we assumed that there should be some room for improvement for PEC management in certain LAGC classes to obtain optimal benefits and personalization and meet the real-world requirement. Interestingly, in the present dataset, patients who had shorter PEC treatment are older, with more comorbidities and poorer physical status. The unbalanced baseline could largely reflect the real-world situations, and the results guide us to understand the true benefit of PEC in such clinical contexts.

**Figure 9:**
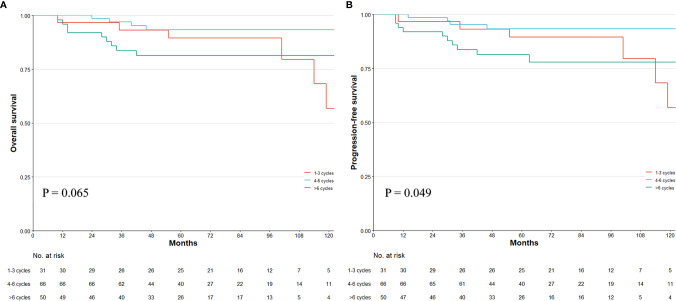
Kaplan–Meier survival plot of the overall survival (OS) and progression-free survival (PFS) in different PEC duration of three tiers in ypT0-2N0 patients. p-value stands for log-rank test. Numbers at bottom indicate patients at risk.

As the topic is a challenging issue, we acknowledge that our study has limitations. First, this is a retrospective study with retrospective bias. Second, the k-fold cross-validation is not the best way in measuring the effectiveness of the model for time series data, although this method has been widely adopted in survival regression (40). The leave-one-out and Monte Carlo cross-validation could be a more reasonable solution, but these were not a built-in function in current decision tree algorithms (41). As recursive partitioning is more systematic in dealing with dichotomous endpoints, using the survival tree method may be challenging. Despite these concerns, the splitting nodes that the tree gave out were reasonable and had clinical significance. Third, the four to six cycles interval for ypT0-2 patients is only partially supported by the non-linear regression, although the PEC’s benefit on OS is more appropriate to be illustrated by non-linear regression than the linear Cox regression. The test for the overall effect of the regression model did not reach a significant level (p = 0.128). This means that the PEC cycles increase can be either non-linear beneficial or non-improved. Because there is still a strong tendency to favor the chemotherapy management in our study (**Figure 9**) and previous research results (10), we suggest that the non-significance should have resulted from our relatively small sample size in the subgroup analysis. Thus, multicenter, large sample studies are needed to validate our findings. Fourth, when considering the NAC response, tumor regression should be considered, but this was ruled out in the preliminary analysis. While 37 cases have missing value in this entry, we found that the TRG (AJCC/CAP criteria) is not a prognostic factor in the rest of 230 ypN0 patients (TRG2-3 vs. TRG0-1, HR, 1.19; 95%CI, 0.84–2.12; p = 0.574). Various reasons can be responsible for this non-significance: (1) TRG only reflects the response to chemotherapy but might be a poor prognosticator for early-stage patents who are not required for intensive drug-based therapy; (2) current TRG classifications do not account for the involvement of lymph nodes, ypN0 with pretreated cN+ diagnosis should be a more direct indication for chemosensitivity; and (3) current cutoff values for TRG criteria may not achieve optimal discriminative ability. Fifth, the aim of our study was to summarize the impact of PEC on survival (to reach the maximum sample volume) so that some decisive factors for survival were not included in the study according to our previous reports (15, 42). The c-index should also increase if more factors were input into the tree model. Finally, yet importantly, external validity is a prerequisite for clinical applicability. However, retrieving data with full entries from other tertiary medical centers in our region is admittedly a difficult undertaking. Both the case reporting form and the study protocol will be redesigned to support our multicenter collaboration and to conduct the external validation.

In conclusion, although ypN0 means promising survival outcome, the AC treatment is still necessary for the PEC modality. Specifically, the reduction in PEC duration could be applied for the ypT0-2N0 stage patients but may not be suitable for higher ypT stages. A multicenter-based study with larger sample sizes is required to validate our results.

## Data Availability Statement

The raw data supporting the conclusions of this article will be made available by the authors upon reasonable request. Requests to access the datasets should be directed to ziyu_li@hsc.pku.edu.cn.

## Ethics Statement

The studies involving human participants were reviewed and approved by The Ethics Committee of Peking University Cancer Hospital. The patients/participants provided their written informed consent to participate in this study.

## Author Contributions

JFJ, YKW and ZNL designed the study. The acquisition of data was conducted by YKW, ZNL, YZ and ZYL. The data analysis and interpretation was performed by ZNL and XJY; ZNL and YKW drafted the manuscript. All authors extensively and critically revised the manuscript before submission.

## Funding

This work was funded by Beijing Municipal Health Commission (DFL20181103 and ZYLX201701).

## Conflict of Interest

The authors declare that the research was conducted in the absence of any commercial or financial relationships that could be construed as a potential conflict of interest.

## Publisher’s Note

All claims expressed in this article are solely those of the authors and do not necessarily represent those of their affiliated organizations, or those of the publisher, the editors and the reviewers. Any product that may be evaluated in this article, or claim that may be made by its manufacturer, is not guaranteed or endorsed by the publisher.
